# Editors’ Book Table

**Published:** 1870-12

**Authors:** 


### Editor's Book Table.

[NOTE.—All works reviewed in the columns of the CHICAGO MEDICAL JOURNAL may be found in the extensive stock of W. B. KEEN & COOKE, whose catalogue of Medical Books will be sent to any address upon request.]

*A Handbook of Medical Microscopy.* By Joseph G. Richardson, M.D., Microscopist to the Pennsylvania Hospital, etc., etc. Philadelphia: J. B. Lippincott & Co.

This little treatise supplies a vacuum in American medical literature, long felt by those members of the profession who have devoted any attention to the subject of microscopic investigation.

The literature of the microscope has been hitherto almost exclusively European, but imperfectly adapted to the medical microscopist, by reason of the extent of the field attempted to be embraced, and still more imperfectly to American students, while the costliness of most of the works has placed them outside of the reach of many earnest workers, who are compelled to study, amongst others, the "bread and butter sciences."

Without meaning to depreciate the unquestionably great value of the works of Carpenter, Beale, Hogg, Gosse and others, they certainly fail to supply exactly the need of the tyro in medical microscopy, who is compelled in acquiring them, in order to get a little that he does want, to purchase much that he does not want, has no use for, and which will only serve to distract his attention from the field, already sufficiently wide, of his legitimate inquiry.

We have examined the little volume of Dr. Richardson with much care, and find it to be comprehensive as regards its subject matter, concise in style, and explicit in statement. Having traveled the road leading to practical microscopy when it was only a by-path with no landmarks to guide our doubtful and devious way, we are in a position to appreciate fully the value of the instructions laid down by the author, and the great advantage to be derived from their careful study by those desirous of following in the same path.

The author seems to understand that the physician needs to become in the shortest possible time familiar with the practical application of the microscope to medical diagnosis, and hence, does not compel him to wade through voluminous treatises upon history, optics, mathematics, geology, or palæontology. He don't once in the whole book say "diatom," that hobby of the dilletanti and bore of utilitarians.

While we must confess a sneaking sympathy with his weakness for high powers, we fear that the majority are against us.

The author is a staunch advocate, indeed the ablest in this country, of Cohnheim's theory of the amæboid movements of white blood cells (leucocytes) and of their essential relation to the inflammatory process, and was the first to direct attention to the "Identity of the white corpuscles of the blood with the salivary mucus and pus corpuscles." The demonstration of this identity should entitle him to the gratitude of every doubter who, like the writer, may have for years been puzzled to detect any difference between them by which they could be distinguished from each other.

But enough has been said of the character and scope of a work which we believe will do more to facilitate the studies of medical microscopists than any within our knowledge, and, as such, we give it our most hearty commendation. W. H.

*The Pathology and Treatment of Venereal Diseases: including the Results of Recent Investigations upon the Subject.* By FREEMAN J. BUMSTEAD, M.D., Professor of Venereal Diseases at the College of Physicians and Surgeons, New York, etc., etc. Third Edition, Revised and Enlarged. With Illustrations. Pp. 704. Philadelphia: Henry C. Lea, 1870.

When the first edition of this work appeared, we wrote of it in terms of high commendation. The present edition shows very careful and industrious revision, many parts having been entirely re-written, portions, formerly controversial but now settled, having been omitted, the text in some instances condensed, and the bulk of the entire work increased sixty-four pages. The modifications prominently to be noticed refer to the treatment of stricture, and the operations of ruptures and internal urethrotomy, chancroid and syphilis, visceral syphilis, and syphilitic affections of the eyes. The latter topic has been delegated especially to Dr. E. G. Loring, whom Dr. Bumstead vouches for as unexcelled and rarely equalled in his specialty. We do not hesitate to rank this book as the best on the subject. It is in our opinion the most correct in doctrine as it is certainly the most practical in its teachings. It is "up to the times"—devoid of obscurity, direct in its methods, and reliable in its therapeutics. Physicians who see but little of venereal disease, should have this work for their enlightenment, and those who see much of it will infallibly find suggestions for daily assistance.

*Practical Anatomy: a Manual of Dissections.* By CHRISTOPHER HEATH, F. R. C. S., Lecturer on Anatomy at the Westminster Hospital, etc., etc. First American from the Second English Edition. Edited, with Additions, by William W. Keen, M.D., Lecturer on Pathological Anatomy in the Jefferson Medical College, etc., etc. Pp. 572. Philadelphia: Henry C. Lea, 1870.

The teacher of Practical Anatomy will find this volume of very great assistance, as it comes nearer to what is wanted when the subject is on the table, than any one of the numerous manuals for the dissecting room it has been our fortune to meet. All of them have savored too much of mere descriptive anatomy. This is really a help to him who is beginning to practice upon the cadaver. It is well illustrated by numerous woodcuts and diagrams, and the text excellently well sets forth exactly what the student is to do and look for at each step of his investigations. The American editor has modified the arrangement of the text so as to have it accord more with the methods adopted in this country. He has also incorporated the results of recent important researches—particularly those of Duchenne, Cleland and Luschka; and almost entirely re-written the directions for dissection of the eye.

The name of the American editor has not previously, to our personal knowledge, been attached to any volume as author or editor, but is familiar to us and many of our readers, as a contributor of many interesting and valuable articles to our valued quarterly contemporary, the *Am. Jour. of Med. Sciences.* In his present task he has developed the possession of abilities, both as an author and editor, which we hope hereafter to see employed still further for the benefit of the profession.

*A Treatise on Physiology and Hygiene, for Educational Institutions and General Readers.* Fully Illustrated. By JOSEPH C. HUTCHISON, M.D., President of the N. Y. Path. Soc., Vice-Pres. of the N. Y. Acad. of Med., Surgeon to the Brooklyn City Hospital, late Pres. of the Med. Soc. of the State of N. Y., etc., etc. New York: Clark & Maynard, Publishers, 5 Barclay Street, 1870. From the Publishers, by S. C. Griggs & Co. $1.60. Pp. 270.

The eminent position of the author of this treatise entitles it to notice, although avowedly compiled for popular use. For this purpose we can freely commend it to teachers as being reliable, and as complete as, perhaps, is necessary to the non-professional student. It is perspicuously written—divided into paragraphs with full-faced captions, and at the foot of each page a series of questions to facilitate the work of the teacher. It occurs to us that more illustrations should have been introduced to assist comprehension of the text.

